# Epidemiology of Multidrug Resistant Infections after Inter-ICU Transfer in India

**DOI:** 10.5005/jp-journals-10071-23103

**Published:** 2019-01

**Authors:** Anirban H Choudhuri, Bhuvna Ahuja, Partha S Biswas, Rajeev Uppal

**Affiliations:** 1,2,4 Department of Anesthesiology and Intensive Care, Jawaharlal Institute of Postgraduate Medical Education and Research, New Delhi, India; 3 Department of Psychiatry, Jawaharlal Institute of Postgraduate Medical Education and Research, New Delhi India

**Keywords:** Inter intensive care unit transfer, Multidrug resistant organisms, Non multidrug resistant organisms

## Abstract

**Background and aims:**

The patients in the intensive care unit (ICU) are often infected with multidrug resistant (MDR) organisms. When they are transferred to other ICUs, they can expand the reservoir of MDR organisms and pose a threat to the infection control program. The present observational study was undertaken to describe the epidemiology and compare the outcome of MDR and non-MDR infections after inter ICU patient transfer.

**Materials and methods:**

A retrospective study was conducted in a cohort of 134 consecutive admitted patients in a tertiary care ICU from other ICUs. The primary objective was to measure the prevalence of MDR and non-MDR infections. The secondary objective was to compare the outcome between MDR and non-MDR group and identify the factors independently associated with mortality for each group.

**Results:**

Among 134 patients, 89 had infections (66.4%) and in 29 (21.6%) were due to MDR organisms. The most common organism was *Klebsiella* in the MDR and *E. coli* in the non-MDR group. There was no difference between the groups in mortality, duration of mechanical ventilation and length of ICU stay. The duration of mechanical ventilation and ICU stay >7 days was independently associated with mortality in the MDR group. No association was found in the non-MDR group.

**Conclusion:**

The study demonstrates a high prevalence of MDR infections after inter ICU transfer. There is no difference in outcome between the groups, but the mortality in the MDR group is independently associated with a longer duration of mechanical ventilation and ICU stay.

**How to cite this article:**

Choudhuri AH, Ahuja B, Biswas PS, Uppal R. Epidemiology of Multidrug Resistant Infections after Inter-ICU Transfer in India. Indian Journal of Critical Care Medicine, January 2019;23(1):1-6.

## INTRODUCTION

The admission of patients in the intensive care unit (ICU) occurs from diverse settings. They include operating rooms, emergency rooms, wards, etc. Some patients are also transferred from the ICUs of other hospitals.^1-3^ The reasons for inter ICU transfer can vary and may occur due to unavailability of specialized departments, expectations of better outcome, financial constraints etc. The Intensive Care over Nation (ICON) audit had found that more than one third of ICU patients develop an infection during their ICU stay and Extended Prevalence of Infection in Intensive Care (EPIC II) study had shown that more than half of the patients admitted in the ICU at any time are likely to harbor infections.^4,5^ Therefore inter ICU patient transfer may cause the spread of organisms which may include multidrug resistant (MDR) pathogens. The propagation of MDR organisms during inter ICU transfer poses a threat for ICU infection control program.^6^

This study was conducted with the aim to describe the epidemiological characteristics of MDR organisms transmitted during inter ICU patient transfer and compare outcome between patients transmitting MDR organisms and non-MDR organisms.

## MATERIALS AND METHODS

An observational, retrospective study was conducted in a cohort of 134 consecutive ICU patients transferred from ICUs of other hospitals to the mixed medical–surgical ICU of a 750 bedded tertiary care superspecialty teaching institute between July 2014 and April 2018. Data were extracted from the ICU database maintained for the administrative and clinical record keeping. Approval was sought from the Institution Ethics Committee for a waiver of informed consent.

The study objective was to measure and compare the epidemiological characteristics, clinical features, organisms grown and outcome of the MDR versus non-MDR infections in patients transferred from other ICUs to our ICU.

The following definitions were used for the study.

MDR infection was considered on the basis of the exis ting knowledge during the study period which included the following pathogens with given antibiotic resistance characteristics: extended-spectrum-lactamase-producing gram-negative Enterobacteriaceae, such as *Klebsiella* spp., *E. coli*, and *Proteus* spp.; *P. aeruginosa* resistant to ceftazidime or carbapenems; other pan-resistant Entero-bacteriaceae bacteria or those sensitive only to carbapenems; *Acinetobacter* spp. resistant to ampicillin, ampicillin/sulbactam, or carbapenems; methicillin-resistant staph aureus (MRSA) and vancomycin-resistant Enterococcus (VRE) spp. Other organisms were considered MDR if they were found to be resistant to at least three of the following antibiotic classes: antipseudomonal cephalosporins/penicillins, macrolides, carbapenems, fluoroquinolones, aminoglycosides, colistin, and tigecycline.

Non-MDR infection–all infections other than those included as MDR infection.

The presence or absence of infection was suspected by the ICU physician during clinical examination. All suspicious samples were sent for microbiological examination on the same day of admission and not later than 24 hours of admission. Only the first microbiological report obtained after ICU admission was considered for determining the microorganisms transmitted from the previous ICU and their sensitivity pattern.

One or more of the following specimens were investigated to identify the microorganisms and determine their antibiotic resistance pattern–blood, urine, endotracheal aspirate (ETA), drain fluid (which includes pleural fluid, peritoneal fluid, and cerebrospinal fluid). The isolates with colony forming unit count (CFU) more than 10^5^/mm^3^ were considered as an infection.

The patients with clinical suspicion of infection but showing no growth of microorganisms were considered noninfectious. The patients with no clinical signs of infection but showing growth of microorganisms were considered as contaminants.

**Graph 1:**
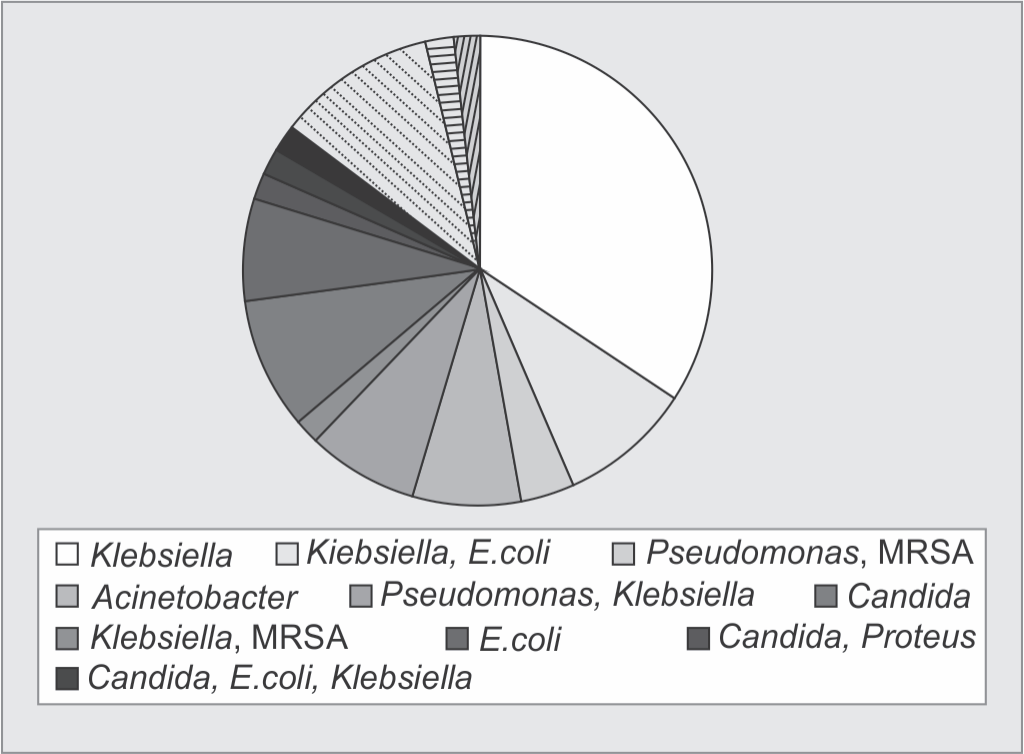
Distribution of organisms in the MDR group

The prevalence of organisms and their distribution in the MDR and non-MDR group were separately measured. The following variables were chosen to compare the outcome of patients with MDR infections and those with non-MDR infections: age, sex, the presence of sepsis, disease severity (APACHE II), diagnosis, number of ventilator days, duration of ICU stay and mortality.

All data were summarized by routine descriptive statistics. 95% confidence interval (CI) values were calculated for key variables. Numerical variables were compared using Student’s independent samples *t* -test when normally distributed or by Mann–Whitney U-test when otherwise. Fisher’s exact test was used for intergroup comparison of independent proportions. Univariate analysis was two-tailed, and p < 0.05 was considered statistically significant. Univariate and adjusted odds ratios (ORs) from the logistic regression analysis were reported. Statistical Package for Social Sciences (SPSS) Statistics version 17 (Illinois, Chicago: SPSS Inc., 2008) software was employed for statistical analysis.

## RESULTS

**Graph 2:**
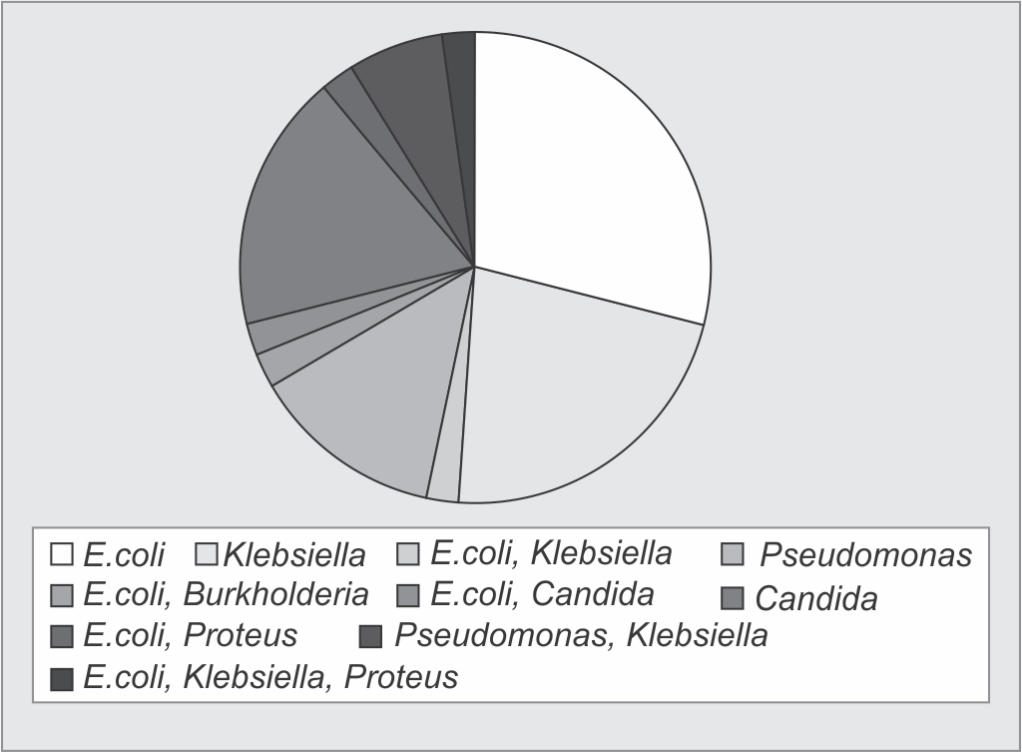
Distribution of organisms in the non-MDR group

Out of 134 patients who were transferred from ICU of other hospitals, 89 patients had infections (66.4%), and 29 patients had infections due to MDR organisms (21.6%). Graphs 1 and 2 show the distribution of organisms in MDR and non-MDR group. It is seen that the majority of organisms identified in the MDR group is *Klebsiella* (68%) followed by *Acinetobacter* (43%) and *E. coli* (37%). In the non-MDR group, the main organisms are *E. coli* (44%), *Klebsiella* (38%) and *Pseudomonas* (32%).

**Graph 3:**
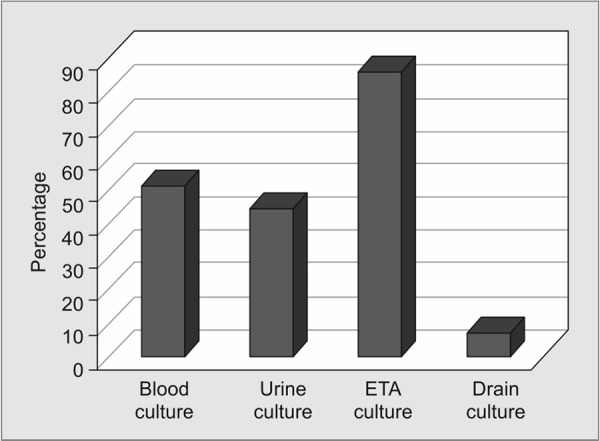
Presence of MDR organisms in the different body fluids. ETA = endotracheal aspirate

**Graph 4:**
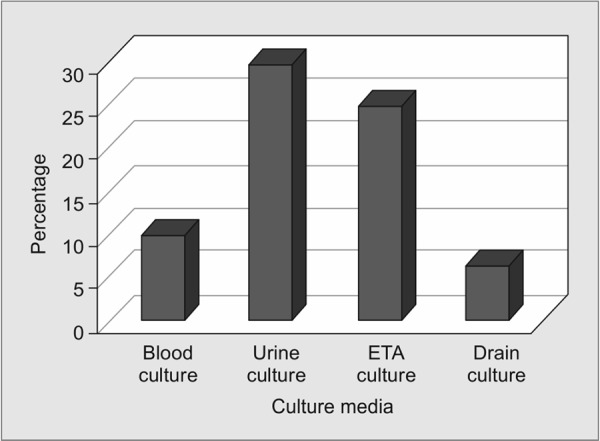
Presence of non-MDR organisms in different body fluids. ETA = endotracheal aspirate

Graphs 3 and 4 show the presence of microorganisms in the different body fluids in MDR and non-MDR group. It is seen that most of the organisms are isolated from the endotracheal aspirate (91%) in the MDR group and urine (29%) in the non-MDR group.

Table 1 shows a comparison of the clinical features and outcome of patients in MDR and non-MDR groups. There is no difference in the two groups with regard to the presence of sepsis (62.1% *vs.* 46.7%; p = 0.17), APACHE II score (20.5 *vs.* 19.4; p = 0.55), number of ventilatory days (7.5 *vs.* 8.6; p= 1.99), number of ICU days (8.7 *vs.* 10.6; p=1.69) and mortality (69% *vs.* 45%; p = 0.94).

Tables 2 and 3 show the results of logistic regression analysis for each factor as an independent cause of mortality in MDR and non-MDR groups. It is found that the duration of ventilatory days greater than 7 days and the duration of ICU stay of more than 7 days are independent causes of mortality in the MDR group. No such association is found in the non-MDR group.

**Table 1:** Comparison of clinical features and outcome of MDR and non-MDR group

		*MDR (n = 29)*	*Non MDR (n = 60)*	*p value*
Age (mean ± SD)		54.41 ± 12.9	52.03 ± 13.8	0.81
Sex (M/F)		16/13	32/28	0.66
Presence of sepsis	Yes	18 (62.1%)	28 (46.7%)	
	No	11 (37.9%)	32 (53.3%)	0.17
APACHE II (mean+/-SD)		20.59 ± 4.8	19.42 ± 4.4	0.55
Diagnosis n (%)	Acute pancreatitis	5 (17.2)	5 (8.3)	
	Acute liver failure	4 (13.8)	3 (5.0)	
	Brain tumor	0	2 (3.3)	
	Ca. colon	0	2 (3.3)	
	Ca esophagus	0	2 (3.3)	
	Ca stomach	0	1 (1.7)	
	Interstitial lung disease (ILD)	3 (10.3)	3 (5.0)	
	Stroke	7 (24.1)	24 (40.0)	
	Perforation peritonitis	0	1 (1.7)	
	Ca gallbladder	5 (17.2)	3 (5.0)	
	Heart failure	1 (3.4)	2 (3.3)	
	Ca liver	0	1 (1.7)	
	Meningitis	0	1 (1.7)	
	Pneumonia	1 (3.4)	1 (1.7)	
	Postoperative leak	3 (10.3)	3 (5.0)	
	Postpartum eclampsia	0	1 (1.7)	
	Postoperative sepsis	0	3 (5.0)	
	Posterior reversible encephalopathy syndrome (PRES)	0	1 (1.7)	
	Traumatic brain injury (TBI)	0	1 (1.7)	0.48
Days on ventilator (mean ± SD)		7.59 ± 5.3	8.62 ± 6.4	1.98
Days in ICU (mean ± SD)		8.72 ± 6.4	10.68 ± 7.9	1.69
Mortality n (%)	Yes	20 (69%)	27 (45%)	0.94
	No	9 (31%)	33 (55%)	

(APACHE– Acute physiology and chronic health evaluation; Ca–Cancer)

**Table 2:** Logistic regression of MDR group

		*Significance*		*95% CI*	
*Factors*	*β value*	*(p value)*	*OR*	*Lower*	*Upper*
Age	0.060	0.290	1.061	0.960	1.185
Sex	–0.043	0.970	0.958	0.104	8.809
APACHE II	–.0194	0.320	0.823	0.561	1.208
Ventilator days >7 days	2.283	0.001	9.802	2.676	35.901
Length of stay in ICU >7 days	–2.299	0.001	3.100	0.029	0.342
Sepsis	–1.504	0.331	0.222	0.011	4.611

(OR–Odds ratio; CI–Confidence interval)

## DISCUSSION

Our study has found a high prevalence of MDR and non-MDR infections after inter ICU patient transfer. This is comparable to the published reports for inter-hospital transfer as specific data for inter ICU transfer is not available in the literature *per se*.^4,5,7,8^ The improvement in patient referral and transport has resulted in an increased patient transfer from one facility to another which is likely to further escalate in the near future.^9,10^ Since a good number of such patients are shifted from the ICU, the risk of infection spread particularly due to MDR organisms is a serious concern. Although some studies have been conducted with the object of identifying preventive strategies for dissemination of MDR pathogens, inadequate epidemiological information has been a major limiting factor in elucidating clear facts and delineating effective strategies.

In our study, the commonest organism isolated in the MDR group was *Klebsiella* while that in the non-MDR group was *E. coli*. Both these organisms belong to the highly virulent ESKAPE group which has gained considerable notoriety over the past few years as the dominant resistant bugs. More alarming and more serious are the progressive rates of their increase in resistance to the newer drugs. The European Antimicrobial Resistance Surveillance Network (EARS-Net) has reported that the combined resistance to three antibiotic groups namely fluoroquinolones, third-generation cephalosporins and aminoglycosides has increased for *Klebsiella* in Europe over the years 2011 to 2014 by four folds.^11-13^ A more worrisome trend has been reported from Italy where carbapenem-resistant (CR) *Klebsiella pneumoniae* which were non-existent in 2008 jumped to 60% in 2013 (CDDEP).^14^ In India, according to the Centre for Disease Dynamics, Economics and Policy (CDDEP) 80% of the *Klebsiella pneumoniae* isolates are resistant to cephalosporins and up to 60% are resistant to carbapenems.^13^ This raises concern for early initiation of prevention strategies during ICU to ICU transfer because of the high mortality associated with certain *Klebsiella* infections.^14^ It also highlights the need for molecular characterization of the strain to understand the mechanisms for resistance in *Klebsiella* isolates before initiating appropriate antimicrobial therapy.

**Table 3:** Logistic regression of non-MDR group

		*Significance*		*95% CI*	
*Factors*	*β value*	*(p value)*	*OR*	*Lower*	*Upper*
Age	–0.672	0.936	0.86	0.654	2.336
Sex	–41.024	0.898	0.91	1.742	5.648
APACHEII	–3.754	0.497	1.05	0.842	2.486
Ventilator days >7 days	45.238	0.466	1.12	0.902	1.794
Length of stay in ICU >7 days	–44.449	0.990	0.94	1.256	7.236
Sepsis	–35.303	0.904	0.76	1.289	9.478

(OR- odds ratio; CI- confidence interval)

The most common pathogen identified in the non-MDR group is *E. coli* which are commonly considered as a commensal organism in the gastrointestinal tract. However, *E. coli* is also known to cause serious infections and death in unfavorable conditions. With increasing resistance to cephalosporins among members of Enterobacteriaceae due to the spreading of extended-spectrum β-lactamases (ESBL) emergence of resistance to most first-line antimicrobial agents has been spreading far and wide.^15,16^ Although patient to patient transmission of *E. coli* is not considered to be common, the acquisition of ESBL producing *E. coli* in any ICU setting can be troublesome.

Among the samples, ETA is considered a reliable alternative to bronchoalveolar lavage (BAL) and protected specimen brush (PSB) in the diagnosis of pneumonia including ventilator-associated pneumonia (VAP). The majority of MDR organisms in our study were recovered from ETA, and hence MDR VAP is an important consideration during inter ICU transfer. Because of diverse resistance determinants among *Klebsiella pneumoniae* causing MDR VAP, it is difficult to predict the strains that are more amenable for transfer in hospitalized patients.^17^ It has been reported that the cumulative risk of VAP increases but the hazard ratio decreases with ICU stay (3.3% at day 5, 2.3% at day 10, and 1.3% at day 15).^18^ Therefore greater the duration of ICU stay, greater is the likelihood of VAP even without an apparent deterioration in the clinical condition of the patient. Therefore it is imperative to formulate strategies and adopt measures to minimize the spread of MDR organisms through ETA which is only possible through specific VAP control measures. On the other hand, the growth of non-MDR organisms in the urine indicates that urinary tract infection is common but amenable to treatment with appropriate antibiotics which may be useful even after transfer to the new ICU.

Our study did not find any difference in outcome in terms of mortality, length of ICU stay and the duration of ventilatory days in patients between the MDR and non-MDR groups. This can be explained by stating that mortality and longer hospital stays were found to occur in patients with late diagnosis and ineffective initial therapy.^18,19^ This did not happen in our patients as the diagnosis was rapidly performed. If rapid diagnostic tests are performed for the identification of MDR organisms and appropriate therapy is instituted early some improvement in the outcomes can be expected. It is for the same reason that the management of patients with MDR bacteremia has been greatly optimized in many centers with the introduction of matrix-assisted laser desorption ionization/time-of-flight mass spectrometry (MALDI TOF MS).^20^ In a retrospective observational study from Ghent, pathogen prediction for bloodstream infection by tracheal surveillance cultures in cases of hospital-acquired pneumonia was associated with a higher rate of adequate empiric antibiotic therapy. This was associated with increased survival in both univariate and multivariate analysis.^21^

Our study found that duration of greater than 7 days for both mechanical ventilation and length of ICU stay were independent predictors of mortality in case of MDR but not for non-MDR organisms. This highlights the elevated risk of adverse outcomes associated with MDR organisms and justifies following stricter screening protocols for all colonizers irrespective of their being MDR or non-MDR. Various studies conducted in this context have shown a positive impact of screening protocols from the outset accompanied by an optimized drug therapy on patient-specific basis.^22-24^ It is perhaps more useful to institute precautionary measures in patients after inter ICU transfers who show positive culture positive for high-risk pathogens. This can consist of a private room, hand washing, gloves/gowns/mask, etc. as may be appropriate for the setting. Furthermore, decontamination methods like chlorhexidine bath may be useful additions in improving care and reducing risk.^25^ Some studies have reported that biofilm production attributes to the resistance by facilitating transfer through plasmids. This has an important bearing on our study results as most of the MDR organisms were recovered from ETA which has the highest probability of biofilm formation.^26,27^ Biofilm not only leads to the development of VAP but also stands as a pathogenic mechanism of resistance and impaired response to treatment. Newer silver-coated, gardinecoated and gendine-coated endotracheal tubes (ETT) have been found to be useful in reducing biofilm production. But their ability in inhibiting biofilm production by all organisms is not proven. The isolation of *Acinetobacter* in a large proportion of the patients in the MDR group indicates its quintessential transformation from a colonizer to a pathogen. It has been reported that the risk factors associated with MDR *Acinetobacter* colonization differ by ICU type and acts as a marker of disease severity and of developing subsequent *Acinetobacter* infection and of dying during hospitalization. Therefore active surveillance is necessary to guide empirical antibiotic selection and inform infection control practices in all *Acinetobacter* infections.^28^ There has also been a spurt of reports on *Acinetobacter baumanii* infection contracted through various health care equipments. These include contaminated ventilator equipments, humidifiers, etc. Therefore, inter-ICU transfer of patients on a ventilator should be preceded by a screening of such equipment and application of filters etc. when appropriate.^29,30^

Our study has certain limitations. One, all the patients have a variable period of ICU stay with some having intervening periods of stay inwards. Therefore the flora isolated at the first instance may not be the best indicative sample of the previous ICU. However, there was minimal delay in collection of samples after arrival in our ICU which can circumvent the odds. Second, the tests performed for the identification of organisms in our laboratory have evolved over the period on which we had no control. However, the standard EUCAST and CLSI guidelines were always followed. Lastly, all the limitations inherent due to the retrospective nature of the study need a cautious interpretation.

To conclude, our study is the first of its kind to describe the epidemiological characteristics of transmission of infection following inter ICU transfer. It shows a high prevalence of both MDR and non-MDR infection transmission after inter ICU transfer. Well designed and multicentric prospective studies are essential to identify the risk factors and describe strategies for containing the transmission.
